# Comprehensive evaluation and screening of drought resistance in maize at germination and seedling stages

**DOI:** 10.3389/fpls.2025.1672228

**Published:** 2025-09-08

**Authors:** Wentao Du, FuLai Zhang, Lei Zhang, Mengting Hu, Huijuan Tian, Ying Hao, Shuqi Ding, Dan Zhang

**Affiliations:** ^1^ College of Agriculture, Tarim University, Alar, China; ^2^ Key Laboratory of Genetic Improvement and Efficient Production for Specialty Crops in Arid Southern Xinjiang of Xinjiang Corps, Alar, China

**Keywords:** maize, germination stage, seedling stage, drought resistance, screening

## Abstract

**Introduction:**

Maize is one of the most important staple crops globally, but drought stress has emerged as a major abiotic factor limiting its productivity.

**Methods:**

This study, conducted in 2023 at the Key Laboratory of Genetic Improvement and Efficient Production of Specialty Crops in Southern Xinjiang Arid Regions, College of Agriculture, Tarim University, aimed to identify key drought-tolerant traits and maize varieties. Drought stress was applied during the germination and seedling stages, and 15 drought resistance indices, including germination rate (GR), germination energy (GE), plumule length, plant height (PH), and chlorophyll content (ChL), were comprehensively evaluated.

**Results:**

Drought stress significantly reduced GR, GE, and the germination index, while also inhibiting coleoptile and radicle growth. It further impeded seedling development, as evidenced by decreased PH, reduced ChL content, and lower biomass accumulation. Correlation analysis showed varying degrees of association among the 15 drought resistance indices. Principal component analysis grouped these indices into five composite components, accounting for 75.22% of the total cumulative variance. Stepwise regression analysis produced the following equation: D = -0.002 + 0.13×GEI + 0.938×RFWI + 0.115×RLI + 0.112×DWPI + 0.097×ChlI. Based on the D values, cluster analysis classified 41 maize germplasm resources into five categories: extremely drought-tolerant, strongly drought-tolerant, moderately drought-tolerant, drought-sensitive, and highly drought-sensitive. The extremely drought-tolerant varieties included Yuanyuan 1, Ximon 6, Jinongyu 309, Nongkeyu 368, Xuanhe 8, Hengyu 369, and Nongfu 99.

**Discussion:**

These findings provide a theoretical foundation for breeding drought-resistant maize and identifying drought-tolerant genotypes.

## 1 Introduction

Maize (*Zea mays* L.), one of the most important staple crops worldwide, serves not only as a major food source for humans but also as a key raw material for livestock feed and industrial applications (Yajing et al., 2024). However, with the intensifying effects of global climate change, drought stress has become one of the primary abiotic factors limiting maize productivity (Wang et al., 2016; Yue et al., 2024). Approximately one-third of the world’s land area consists of arid and semi-arid regions (Fang and Xiong, 2014). Xinjiang, a representative arid and semi-arid maize-growing region in northwest China (Li et al., 2017; Cheng and Yin, 2021), has experienced increasingly frequent high-temperature and drought events in recent years. These extreme climatic conditions have severely impacted maize yield and quality, causing harvest losses of up to 50% (Fisher et al., 2015). Consequently, the efficient screening of drought-tolerant maize germplasm has become strategically important for ensuring stable maize production in arid and semi-arid areas, optimizing land-use efficiency, and addressing the challenges posed by climate change.

The germination and seedling stages are critical periods in maize development, during which the crop is particularly sensitive to drought (Ahmad et al., 2009; Huang and Song, 2013). Drought exposure during these stages significantly hampers seed germination, resulting in lower emergence and seedling survival rates (Xiaoyan et al., 2020), while also restricting coleoptile elongation, root development, and water uptake (Tripathi et al., 2024). Prolonged drought stress further suppresses overall plant growth, causing marked reductions in biomass accumulation (Efeoğlu et al., 2009) and disrupting photosynthetic processes, such as chlorophyll synthesis, ultimately reducing yield potential (Yang et al., 2023). These findings underscore the importance of early-stage growth in determining final productivity, making the evaluation of drought resistance during germination and seedling stages essential for enhancing drought resilience and ensuring food security (Bao et al., 2023).

In recent years, various traits associated with drought tolerance at the germination and seedling stages have been widely investigated. Key indicators such as germination rate (GR), germination vigor, radicle length (RL), coleoptile length, plant height, and chlorophyll content (ChL) are commonly used to screen drought-resistant maize genotypes (Ghebremariam et al., 2013; Carvalho et al., 2019; Wang and Peng, 2021). During germination, traits like GR, vigor, and germination index (GI) directly reflect seed viability under drought conditions and serve as primary screening criteria for distinguishing varietal emergence potential (Cheng et al., 2017). Radicle and coleoptile lengths influence seedling anchorage and water absorption efficiency, respectively (Jin et al., 2013; Badr et al., 2020), while radicle number correlates with the spatial capacity for water uptake (Singh et al., 2010). At the seedling stage, plant height and biomass accumulation provide visual indicators of growth inhibition under drought (Wang et al., 2023), whereas Chl reflects photosynthetic capacity and is often used as a proxy for drought resistance (Arunyanark et al., 2008; Qi et al., 2021). Additionally, drought-tolerant genotypes typically increase root biomass to enhance water acquisition (Poorter et al., 2011). This multidimensional trait system, encompassing morphological, physiological, and growth-related parameters, allows for more comprehensive evaluations than single-trait assessments, thereby improving screening accuracy.

Germplasm screening is foundational to crop breeding (Guohong et al., 2025). The in-depth exploration and efficient use of genetic resources not only provide diverse parent materials for drought-resistance breeding but also accelerate the development of climate-resilient maize varieties (Yu et al., 2024). Cultivating highly drought-tolerant genotypes reduces irrigation demands and agricultural costs (Fita et al., 2015; Rosero et al., 2020), and drought tolerance remains a critical trait for successful cultivation in arid zones (Cooper et al., 2014). However, current screening methods, especially those applied during the sensitive germination and seedling stages, face three major limitations: (1) Drought tolerance is a complex polygenic quantitative trait, and traditional phenotyping methods are labor-intensive and inefficient, limiting large-scale screening efforts (Liu et al., 2023); (2) Evaluations based on single or limited traits often fail to capture the comprehensive drought response under field conditions, leading to inaccurate conclusions (WU and BAO, 2012; Li et al., 2023); and (3) There remains an urgent need to identify efficient core traits for streamlining drought-resistance screening protocols. Although multivariate statistical methods, such as principal component (PC) analysis (PCA) (Jie et al., 2020), membership function analysis (Cheng et al., 2017), and stepwise regression (Saed-Moucheshi et al., 2013), have shown promise in dimensionality reduction and trait selection, systematic evaluations integrating multiple traits across both developmental stages, particularly in maize germplasm from arid northwestern regions, are still lacking and require further study.

Field-based drought-resistance screening under real-world arid conditions remains highly valuable (Mohi-Ud-Din et al., 2021). However, the unpredictability of natural precipitation makes it difficult to simulate consistent drought stress in field trials (Muscolo et al., 2013). In contrast, polyethylene glycol (PEG - 6000)-induced osmotic stress provides a highly controlled and reproducible method for simulating drought at the germination stage (Wu et al., 2021; Mustamu et al., 2023), producing physiological responses similar to those caused by natural drought.Therefore, the present study aimed to: (1) Focus on the drought-sensitive germination and seedling stages; (2) Systematically evaluate 15 drought-resistance traits (e.g., GR, germination vigor, coleoptile length, plant height, Chl) across 41 maize genotypes under simulated drought conditions (20% PEG - 6000 for germination; pot-based water withholding for seedlings) using multivariate statistical analysis; (3) Identify core evaluation traits for early-stage drought resistance; and (4) Screen elite drought-tolerant germplasm through integrated assessment. The findings of this study will provide essential germplasm resources and a scientific basis for the development of drought-resilient maize varieties suited to arid environments.

## 2 Materials and methods

### 2.1 Materials

A total of 41 maize varieties widely promoted in production were selected as test materials in this study. The specific information is presented in **Table 1**.

**Table 1:** Information of tested maize varieties.

Number	Name	Source	Number	Name	Source
V1	Wofeng 188	Shanxi	V22	Xianghe 9918	Liaoning
V2	Youqi 909	Jilin	V23	Fuyu 109	Sichuan
V3	Ximon 208	Ningxia	V24	Xianyu 335	Liaoning
V4	Ximon 3358	Inner Mongolia	V25	Ximon 668	Inner Mongolia
V5	Hongxing 528	Jilin	V26	Linyu 1339	Yunnan
V6	Jixing 218	Jilin	V27	Huxin 338	Liaoning
V7	Jinfengjie 607	Liaoning	V28	Nongkeyu 368	Beijing
V8	Jinongyu 309	Henan	V29	Sanmeng 9599	Liaoning
V9	Jin’ai 588	Inner Mongolia	V30	Yuanyuan 1	Yunnan
V10	Nongfu 99	Inner Mongolia	V31	Xuanhe 8	Yunnan
V11	Zhongxing 618	Inner Mongolia	V32	Qunce 888	Sichuan
V12	Hongxing 990	Jilin	V33	Yuhe 536	Henan
V13	Ping’an 1523	Jilin	V34	Ximon 6	Ningxia
V14	Hengyu 369	Jilin	V35	Bixiang 809	Beijing
V15	Xinnong 008	Inner Mongolia	V36	Huxin 712	Liaoning
V16	Wugu 568	Gansu	V37	Wofeng 9	Shanxi
V17	Zengyu 157	Jilin	V38	Ganxin 2818	Gansu
V18	Xingnong No.1	Sichuan	V39	Xinyu 81	Xinjiang
V19	Fengtian 14	Inner Mongolia	V40	Xinyu 66	Liaoning
V20	Deyu 3000	Gansu	V41	Xinyu 24	Xinjiang
V21	Huiyu 3000	Hebei			

### 2.2 Experimental design for germination stage

In May 2023, a maize seed germination experiment was conducted at the Key Laboratory of Genetic Improvement and Efficient Production of Specialty Crops in the Arid Regions of Southern Xinjiang, College of Agriculture, Tarim University. The experiment employed a constant-temperature incubator (Labotery Artificial Climate Chamber, Tianjin, China), using a 20% PEG - 6000 solution to simulate drought stress. To prepare the solution, 200 g of PEG - 6000 powder was dissolved in 800 mL of distilled water, stirred until fully dissolved, and then diluted to a final volume of 1000 mL. Distilled water was used as the control. Incubation conditions were maintained at a light intensity of 5500 Lux, relative humidity of 60%, and a temperature of 25 °C. Prior to sowing, uniformly sized seeds were selected, sterilized in 75% ethanol for 3 minutes, rinsed three times with distilled water, and then prepared for germination. Two layers of filter paper were placed at the bottom of each germination box (12 cm × 12 cm × 5 cm), and 30 seeds were evenly arranged per box. Each variety was replicated five times. The control group received 40 mL of distilled water per box, while the treatment group received 40 mL of the 20% PEG - 6000 solution, ensuring the liquid level reached halfway up the seeds. To maintain consistent volume and prevent changes in concentration due to evaporation, the respective solutions were replenished daily.

### 2.3 Experimental design for seedling stage

The seedling-stage experiment was conducted from June to August 2023 at the Key Laboratory of Genetic Improvement and Efficient Production of Specialty Crops in the Arid Regions of Southern Xinjiang, College of Agriculture, Tarim University. Drought stress was applied using a pot-based water-withholding method. The cultivation substrate comprised a 1:2 mixture of nutrient soil and local sandy loam, which was sieved and loaded into pots (bottom diameter: 32 cm, height: 35 cm) at a rate of 3.5 kg per pot, followed by quantitative weighing. Before sowing, all pots were uniformly saturated with water to reach field capacity (28.5%) and left to stabilize for 24 hours. Ten seeds were sown in each pot at a depth of approximately 3 cm. Each maize variety was planted in six pots and divided into two treatment groups: normal irrigation and drought stress. In the normal irrigation group, 500 mL of water was applied every two days after the three-leaf-one-heart stage, maintaining substrate moisture at 70% ± 5% of field capacity. In the drought stress group, irrigation was withheld after the three-leaf-one-heart stage. Substrate moisture was monitored using the weighing method, and when moisture declined to 30% ± 3% of field capacity (typically around the fifth day of withholding), this level was maintained throughout the stress period. If excessive water loss occurred, minimal hydration (no more than 50 mL per application) was administered using a sprayer. After 10 days of drought treatment, five uniformly growing seedlings were selected from each group for the measurement of relevant physiological and morphological traits.

### 2.4 Project measurement

Germination energy (Equation 2) potential was recorded on the 4th day after sowing, and GR was assessed on the 7th day (Equation 1). The GI was also calculated (Equation 3). On the 7th day, uniformly growing seedlings were selected for sampling. Buds and roots were then separated to measure and record plumule length (PL), RL, and radicle number (Sharifi and Peyman, 2010). The fresh weights of plumules and radicles were measured immediately, while dry weights were determined by first killing the samples at 105 °C in an oven, followed by drying at 80 °C to a constant weight (Yao et al., 2019).


(1) Germination rate (%)=(number of germinated seeds/number of tested seeds)×100%

(2) $$\begin{array}{c} \text{Germination energy }(\%)=(\text{number of germinated seeds within}/\\ \text{a specific periodtotal number of test seeds})\times 100\% \end{array}$$

(3) $$\text{Germination index }(\%)=1.00\times {\text{nd}}_{2}+0.75\times {\text{nd}}_{4}+0.50\times {\text{nd}}_{6}+0.25\times {\text{nd}}_{8}$$

Where nd_2_, nd_4_, nd_6_, and nd_8_ denote the germination rates on days 2, 4, 6, and 8, respectively.

Seedling-stage measured traits: Following the drought stress treatment, five uniformly growing seedlings from each variety were selected for analysis. Plant height was measured first using a measuring tape (Shi et al., 2020). Subsequently, the SPAD value of the leaves was recorded at the widest point of fully expanded leaves using a SPAD - 502Plus portable chlorophyll meter (Konica Minolta, Tokyo, Japan) (Avramova et al., 2016). The seedlings were then carefully uprooted, washed, and separated into aboveground and underground parts. Fresh weights of both parts were immediately measured using an electronic balance with a precision of 0.001 g. The samples were then placed in kraft paper bags, deactivated in an oven at 105 °C for 30 minutes, and dried at 80 °C until a constant weight was reached. Finally, the dry weights of the aboveground and underground parts were measured using the same precision electronic balance (Yao et al., 2019).

### 2.5 Data processing

The methods described by (Ming et al., 2025) and (CaiJin et al., 2025) were used to evaluate the drought resistance of different maize varieties using the Drought Resistance Index (DRI) and the comprehensive evaluation index (D-value). The calculation formulas are as follows:


(4) $$DRC=\frac{Ti}{CKi}$$

where T and CK represent the trait determination values under drought stress and normal water supply conditions, respectively.


(5) $$DRI=DRC\frac{Ti}{Ti_{mean}}$$

where T and T_mean_ represent the trait determination value under drought stress and the average trait determination value of all varieties under drought stress, respectively.

The membership function values were calculated using the following formula:


(6) $$\text{U}(Xi)=\frac{Xi{\text{-Xi}}_{\text{min}}}{Xi_{\max}-Xi_{\min}}$$

where Xi sub denotes the i-th comprehensive index; and Xi_min_ and Xi_max_ represent the minimum and maximum values of the i-th comprehensive index, respectively.


(7) $$Wi=\frac{Pi}{\sum_{i=1}^{n}Pi}$$

where Wi denotes the weight of the i-th index among all indices, and Pi represents the contribution rate of the i-th comprehensive index.


(8) $$D=\sum_{i=1}^{n}\text{[U}(Xi)\times Wi]$$

where the D-value reflects the comprehensive drought resistance under drought stress.

The Drought Resistance Coefficient (DRC) directly reflects the sensitivity of specific traits to drought stress. In contrast, the DRI, by incorporating group comparisons, not only indicates the relative stability of traits but also captures their superior performance under stress conditions. Therefore, DRI is more suitable than DRC for comprehensive evaluation, ranking, and screening across multiple varieties. In this study, DRI was employed for correlation analysis, PCA, and stepwise regression analysis. Finally, cluster analysis based on the D-value was conducted to evaluate drought resistance among the tested maize varieties.

### 2.6 Data analysis and visualization

#### 2.6.1 Descriptive statistics

The minimum, maximum, mean, standard deviation, and coefficient of variation (CV) for germination- and seedling-stage traits (e.g., GR, RL, Chl) were calculated using IBM SPSS Statistics 27 (Armonk, NY, USA) to characterize trait distributions under both control and drought stress conditions (Wang et al., 2023).

#### 2.6.2 Analysis of variance

Multivariate ANOVA was conducted using IBM SPSS Statistics 27 (Armonk, NY, USA) to assess the effects of cultivar, treatment, and their interaction. Statistical significance was determined using the F-test (Tang et al., 2021).

#### 2.6.3 Correlation analysis

Correlation analysis was performed using the Correlation Plot plugin in Origin 2024 (OriginLab Corp., Northampton, MA, USA). Pearson’s correlation coefficients were calculated, with significance levels set at α = 0.05 and α = 0.01 (two-tailed test). The results were visualized as a heatmap displaying both correlation coefficients and significance levels (Zheng et al., 2023).

#### 2.6.4 PCA

PCA was performed using IBM SPSS Statistics 27 (Armonk, NY, USA). PCs_ with eigenvalues >1 were extracted, and varimax rotation was applied to enhance factor loading interpretability (Song et al., 2023).

#### 2.6.5 Stepwise regression analysis

The D-value was used as the dependent variable, while eight drought resistance indices with high factor loadings from the PCA were selected as independent variables. Model fitness was assessed using the coefficient of determination (R²) and adjusted R² ² (Zhan et al., 2013).

#### 2.6.6 Cluster analysis

Cluster analysis was performed in Origin 2024 (OriginLab Corp., Northampton, MA, USA) using Euclidean distance to evaluate varietal differences in drought resistance. The unweighted pair-group method with arithmetic mean was applied to enhance clustering stability. Based on dendrogram thresholds and observed drought resistance performance, cultivars were classified into five categories (Yan et al., 2000).

#### 2.6.7 Membership function analysis

The comprehensive evaluation D-value was calculated using Microsoft Excel 2021 to quantify overall drought resistance capacity (Saeidnia et al., 2023).

## 3 Results and analysis

### 3.1 Analysis of the effects of variety, treatment, and their interaction on maize traits

To elucidate the effects of varietal differences, drought stress treatment, and their interaction on maize germination and seedling traits, an ANOVA was conducted. The results (**Table 2**) showed that both maize variety and drought stress treatment had highly significant effects (p < 0.01) on all germination- and seedling-stage traits. Furthermore, the interaction between variety and treatment also exhibited significant or highly significant differences across all traits. These findings indicate that drought stress markedly inhibits seed germination and early seedling growth in maize. At the same time, clear varietal differences in stress responses were identified, providing a crucial basis for the subsequent screening of drought-tolerant germplasm. The consistently significant variety × treatment interactions highlight the importance of a comprehensive, multi-trait evaluation approach for accurate identification of drought-resistant genotypes.

**Table 2:** Analysis of variance for different maize varieties under drought stress.

Trait	Variety	Treatment	Variety×treatment
GP	22.548**	77.194**	3.27**
GR	40.439**	564.543**	2.541**
GI	29.898**	42.862**	6.388**
PL	10.781**	356.97**	4.455**
RL	10.274**	2259.467**	9.649**
FWP	7.951**	1345.044**	7.474**
DWP	8.617**	617.767**	5.807**
FWR	9.566**	386.591**	2.475**
NR	4.814**	100.403**	2.232**
PH	13.538**	587.133**	4.422**
Chl	6.447**	278.146**	4.163**
SFW	14.947**	700.489**	4.89**
RFW	21.27**	249.185**	3.253**
SDW	22.135**	324.29**	3.815**
RDW	8.363**	132.625**	1.468*

PL, plumule length; RL, radicle length; NR, number of radicles; FWP, fresh weight of plumule; FWR, fresh weight of radicle; DWP, dry weight of plumule; SFW, shoot fresh weight; RFW, root fresh weight; SDW, shoot dry weight; RDW, root dry weight; GR, germination rate; GP, germination potential; GI, germination index; PH, plant height; Chl, chlorophyll content.

### 3.2 Effects of drought stress on seed germination-related traits

**Figure 1:**
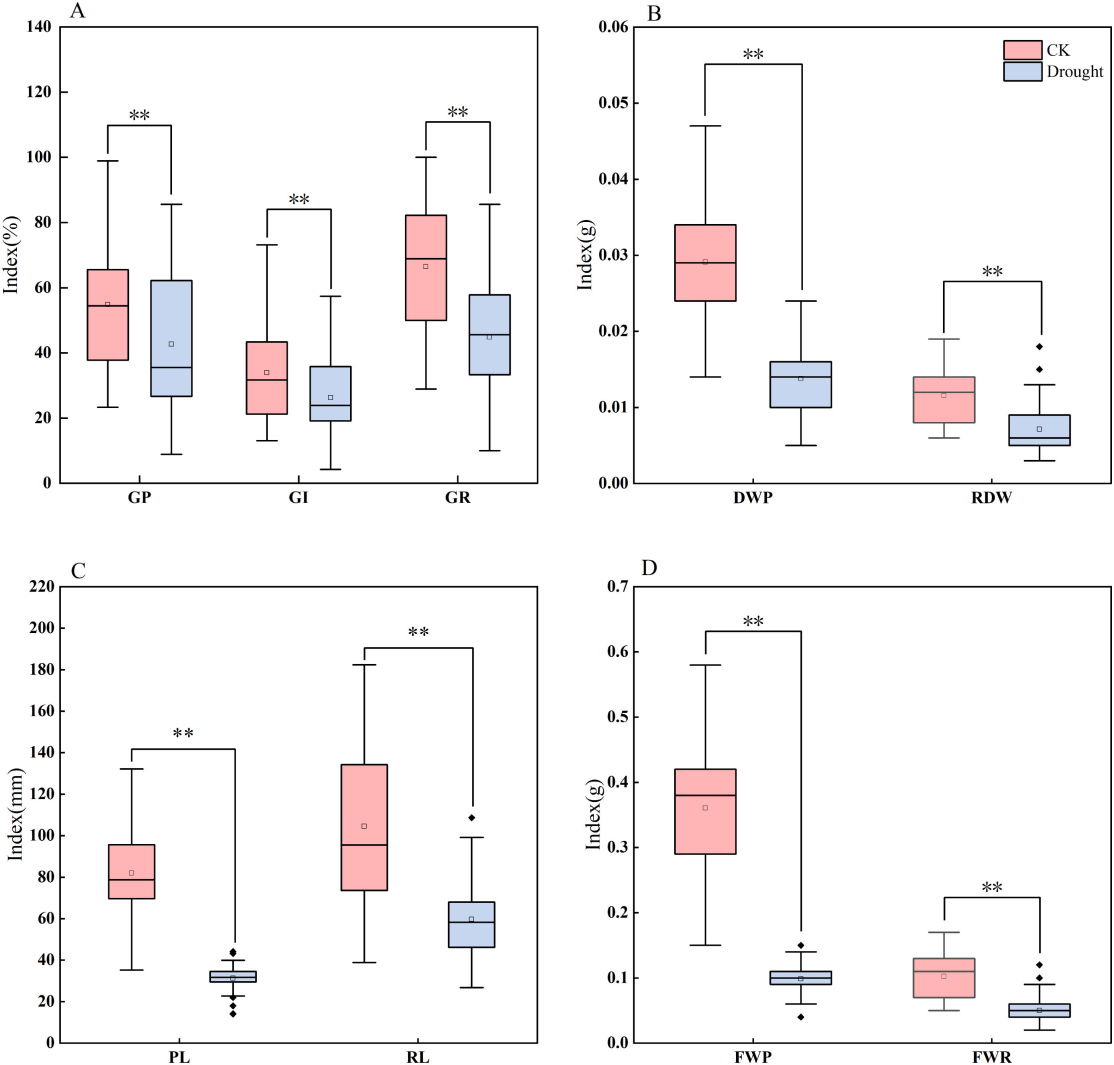
Boxplots of different traits in maize under drought stress and normal water supply. The four groups of boxplots **(A–D)** show different indices (parameters related to GR, DWP, RL/PL, and FWR) under CK (control) and drought stress, respectively. GR, germination rate; GP, germination potential; GI, germination index; DWP, plumule dry weight; RDW, root dry weight; PL, plumule length; RL, radicle length; FWP, plumule fresh weight; FWR, radicle fresh weight. ** indicates statistical significance at the 0.01 level.

Based on the distribution characteristics observed in the box plots (**Figure 1**) and the results of descriptive statistical analysis (**Table 3**), this study found that drought stress simulated by 20% PEG - 6000 significantly inhibited maize seed germination and biomass accumulation. The inhibitory effects were reflected in reduced germination vigor, including declines in GE, GR, and GI, as well as suppressed radicle and plumule elongation, indicated by decreased RL and PL. Additionally, biomass accumulation was negatively affected, as evidenced by reductions in plumule fresh weight (PFW), plumule dry weight (PDW), and radicle fresh weight (RFW). Notably, RL, PL, and DWP showed the most severe reductions, each exceeding 60%, suggesting that these traits are particularly sensitive to drought stress during the germination stage.

**Table 3:** Descriptive statistical analysis of maize germination traits.

Process	Trait	Min	Max	Mean	SD	CV%
CK	GR	28.89	100.00	66.48	19.31	29.05
	GP	23.33	98.89	54.80	20.65	37.69
	GI	13.08	73.17	33.93	15.38	45.33
	RL	38.82	182.39	104.47	37.29	35.70
	PL	35.20	132.16	81.91	20.56	25.10
	FWP	0.15	0.77	0.38	0.14	35.11
	FWR	0.05	0.23	0.10	0.04	36.10
	DWP	0.01	0.06	0.03	0.01	37.89
	NR	3.60	13.40	9.01	2.09	23.16
Drought	GR	10.00	85.56	44.85	18.90	42.13
	GP	8.89	85.56	42.66	22.51	52.78
	GI	4.25	57.42	26.24	13.12	49.99
	RL	26.75	108.68	59.68	19.10	32.00
	PL	14.06	44.15	31.29	6.35	20.31
	FWP	0.03	0.17	0.10	0.03	29.83
	FWR	0.02	0.12	0.05	0.02	42.90
	DWP	0.01	0.02	0.01	0.00	35.39
	NR	4.00	12.00	6.77	1.70	25.13

PL, plumule length; RL, radicle length; NR, number of radicles; FWP, plumule fresh weight; FWR, radicle fresh weight; RADW, radicle dry weight; DWP, plumule dry weight; GR, germination rate; GP, germination potential; GI, germination index.

### 3.3 Effects of drought stress on seedling growth and substance accumulation

**Figure 2:**
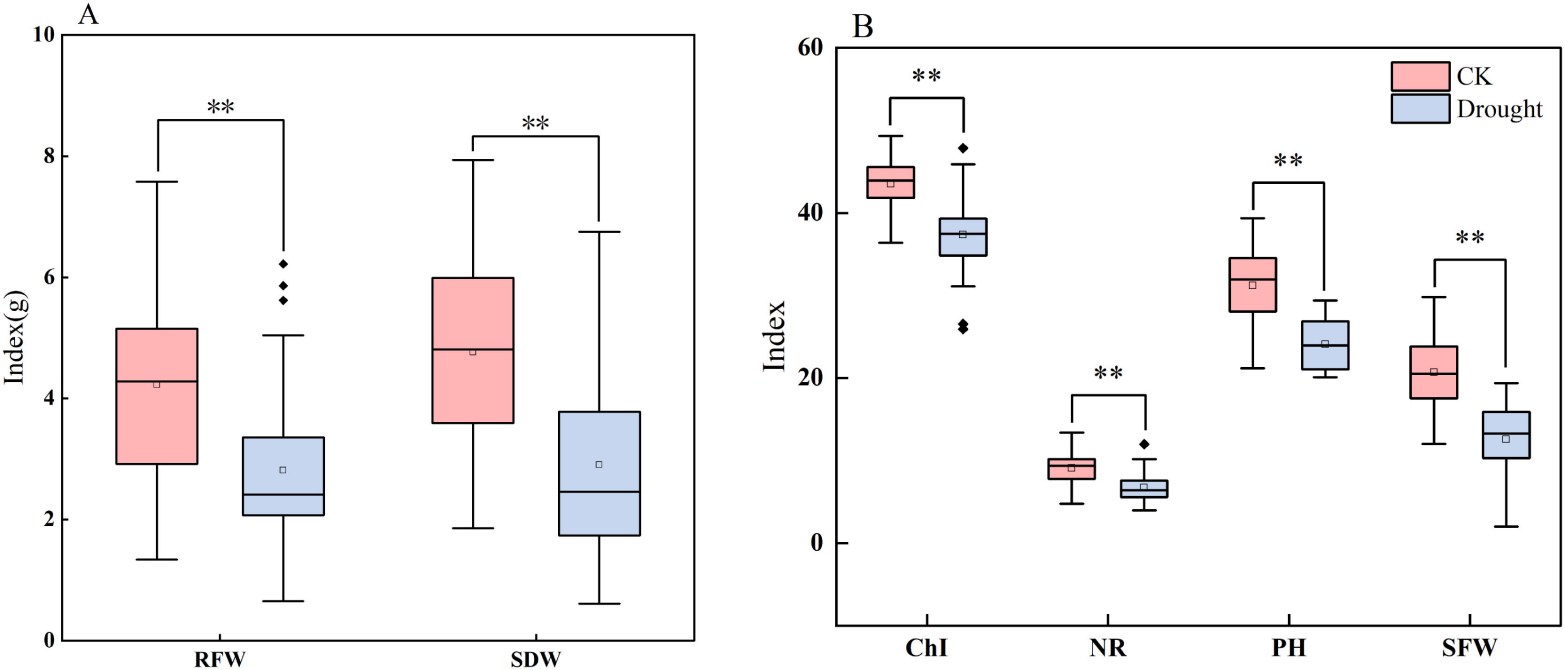
Boxplots of different traits under drought stress and normal water supply. **(A, B)** are boxplots showing the distribution characteristics of different traits (NR, RFW, SDW, RDW, SFW, PH, Chl, etc.) in the control group (CK) and treatment group (Drought), respectively. The boxplots reflect the median and outliers of the data, and are used to compare and analyze the effects of CK and Treat conditions on various traits. RFW, root fresh weight; SDW, shoot dry weight; PH, plant height; Chl, chlorophyll content; NR, number of radicles; SFW, shoot fresh weight. ** indicates statistical significance at the 0.01 level.

To elucidate the profound inhibitory effects of drought stress on the maize seedling growth and development of maize seedlings, this study compared the performance of key seedling traits under normal irrigation versusand drought conditions simulated through pot-based water withholding to simulate drought conditions. Based on boxplotbox plot analysis (**Figure 2**) and descriptive statistics (**Table 4**), comprehensive negative impacts of drought stress on maize seedling growth were observed: a significant suppression ofwas found to significantly impair seedling performance. Specifically, it suppressed plant height increment, reduction in chlorophyll content, inhibition of, reduced Chl, inhibited biomass accumulation (both fresh weight and dry weightweights), and decreased radicle number. Notably, drought stress not only reducedlowered the mean values of these traits but also universally and significantly increased their coefficients of variation (CV). This indicates, indicating substantial genetic variationvariability in drought response among maize varieties during the seedling stage, with certain. Some genotypes exhibiting demonstrated a superior capacityability to maintain relative growth under stress. Among all evaluated traits, biomass-related parameters (, particularly shoot dry weight (SDW) and root dry weight (RDW) demonstrated), exhibited the most pronounced increases in CV, highlighting their potential as criticalkey indicators for screeningidentifying drought-resistant genotypes at the seedling stage.

**Table 4:** Descriptive statistical analysis of different traits in maize at the seedling stage.

Process	Trait	Min	Max	Mean	SD	CV%
CK	PH	21.18	39.34	31.19	4.62	14.82
	ChI	36.40	49.32	43.48	3.17	7.30
	SFW	12.04	29.82	20.68	4.49	21.72
	RFW	1.34	7.58	4.22	1.53	36.29
	SDW	1.86	7.94	4.77	1.60	33.66
	RDW	0.39	2.70	1.38	0.57	40.96
	NFR	14.20	26.00	21.13	3.25	15.38
Drought	PH	20.12	29.40	24.09	3.21	13.31
	ChI	25.94	47.84	37.35	4.40	11.78
	SFW	2.01	19.41	12.55	4.26	33.98
	RFW	0.65	6.22	2.81	1.29	45.88
	SDW	0.61	7.75	2.93	1.72	58.84
	RDW	0.23	1.94	0.82	0.40	48.98
	NFR	10.00	20.80	16.15	2.85	17.66

SFW, shoot fresh weight; RFW, root fresh weight; SDW, shoot dry weight; RDW, root dry weight; PH, plant height; ChI, chlorophyll content; NFR, number of fibrous roots

### 3.4 Correlation analysis of traits in maize at germination and seedling stages under drought stress and normal water supply

**Figure 3:**
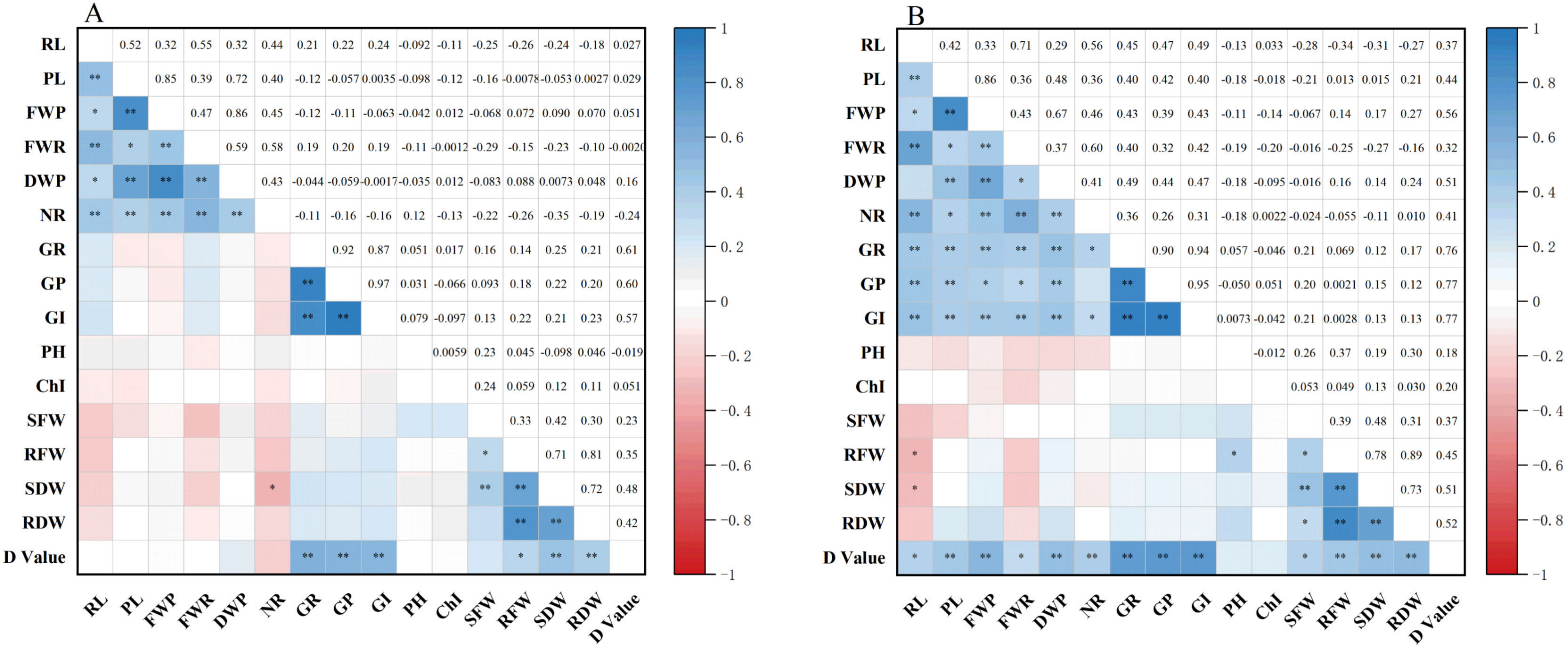
Correlation analysis of different traits under drought stress and normal water supply. * and ** indicate significance at the 0.05 and 0.01 levels, respectively. PL, plumule length; RL, radicle length; NR, number of radicles; FWP, plumule fresh weight; FWR, radicle fresh weight; DWP, plumule dry weight; SFW, shoot fresh weight; RFW, root fresh weight; SDW, shoot dry weight; RDW, root dry weight; GR, germination rate; GE, germination energy; GI, germination index; PH, plant height; ChI, chlorophyll content.

Correlation analysis was conducted to evaluate the relationships between 15 traits, measured at both germination and seedling stages, and the D-value under two conditions: drought stress and normal water supply (**Figure 3**). Under normal conditions, the D-value was significantly or highly significantly positively correlated with six traits: GR, GE, GI, root fresh weight (RFW), SDW, and RDW. These findings indicate that under drought conditions, seed germination vigor (GR, GE, GI), early root development (RL, NR), and seedling biomass accumulation are the key phenotypic components of drought resistance in maize. Notably, six traits, GR, GE, GI, RFW, SDW, and RDW, were significantly correlated with the D-value across both treatments, underscoring seed germination capacity and root performance as core drought-resistance indicators during early developmental stages. Furthermore, the observed correlations among all traits under drought stress reveal underlying physiological interconnections. This highlights the limitation of single-trait assessments and reinforces the need for a comprehensive, multi-trait approach to accurately identify drought-tolerant maize genotypes.

### 3.5 Correlation analysis of drought resistance indices for various traits among different maize varieties

**Figure 4:**
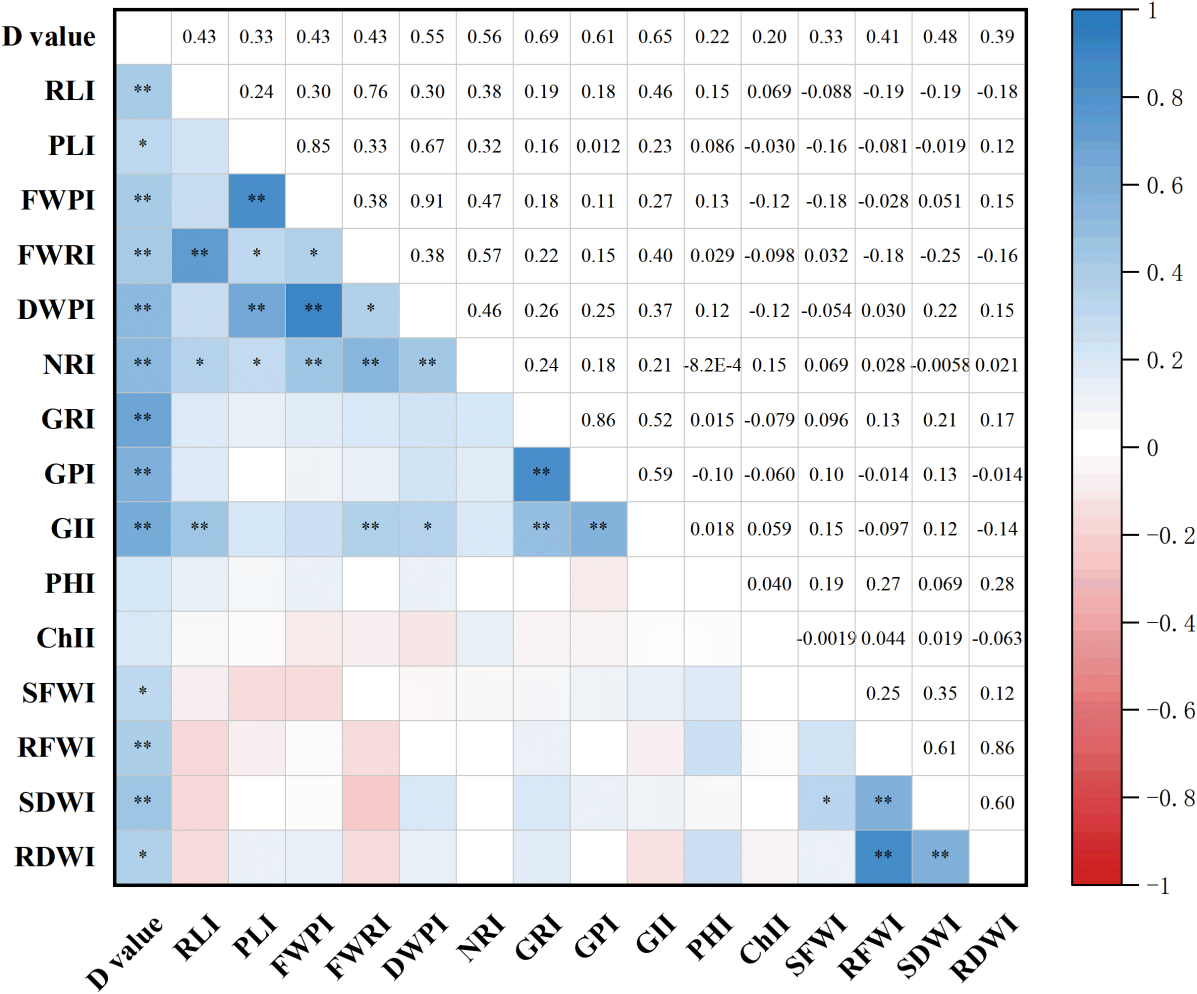
Correlation analysis of drought resistance indices for different traits. * and ** indicate significance at the 0.05 and 0.01 levels, respectively. I, drought resistance index; PL, plumule length; RL, radicle length; NR, number of radicles; FWP, plumule fresh weight; FWR, radicle fresh weight; DWP, plumule dry weight; SFW, shoot fresh weight; RFW, root fresh weight; SDW, shoot dry weight; RDW, root dry weight; GR, germination rate; GP, germination energy; GI, germination index; PH, plant height; Chl, chlorophyll content.

The drought resistance indices for 15 maize traits were calculated using Equations 4, 5 described in section 2.5, and their correlations were analyzed (**Figure 4**). The results revealed several significant or highly significant positive correlations among traits. Specifically, RL index (RLI) was significantly or highly significantly positively correlated with GI (GII), NR index (NRI), and PFW index (PFWI). PL index (PLI) was significantly or highly significantly positively correlated with NRI, PDW index (PDWI), RFW index (RFWI), and FWPI. FWPI also showed significant or highly significant positive correlations with NRI, DWPI, and FWRI. FWRI was significantly or highly significantly positively correlated with GII, NRI, and DWPI, while DWPI was significantly or highly significantly correlated with GII and NRI. GR index (GRI) showed a highly significant positive correlation with GII and germination potential index (GPI), and GPI was also highly significantly correlated with GII. In addition, SFW index (SFWI) was significantly positively correlated with seedling dry weight index (SDWI), while RFWI showed a highly significant positive correlation with both RDW index (RDWI) and SDWI. SDWI and RDWI were also highly significantly positively correlated. These findings indicate that complex interactions among various traits contribute to the drought resistance of maize, highlighting the importance of integrative trait analysis for accurate assessment of drought tolerance.

### 3.6 PCA of drought resistance indices in different maize varieties

#### 3.6.1 Kaiser–Meyer–Olkin and Bartlett’s Sphericity Test

The KMO test and Bartlett’s test of sphericity were conducted on the drought resistance indices of 15 traits (**Table 5**). The KMO value was 0.583, indicating a moderate level of correlation among the variables and confirming that the data were suitable for PCA. Bartlett’s test of sphericity yielded an approximate chi-square value of 378.819 with 105 degrees of freedom and a significance level of p < 0.001, indicating strong correlations among the variables. Together, these results confirmed that the dataset was appropriate for exploratory analyses such as PCA, enabling the identification of potential underlying structures through dimensionality reduction.

**Table 5:** KMO and Bartlett’s sphericity test.

Inspection	Index	Value
Kaiser-Meyer-Olkin measurement value	statistics	0.583
Bartlett’s sphericity test	Approximate Chi-squared value	378.819
	Degrees freedom	105
	Significance	0

#### 3.6.2 PC matrix and total variance explained

PCA was conducted on the drought resistance indices of 15 traits, and five PCs were extracted based on the criterion of eigenvalues greater than 1 (**Table 6**). PC1, with an eigenvalue of 4.03 and a contribution rate of 23.89%, showed high positive loadings on DWPI and FWPI, indicating that this component primarily reflects seed GE reserves and early growth stability. PC2 had an eigenvalue of 2.81 and accounted for 18.70% of the variance, with high positive loadings on RFWI and RDWI, suggesting that this component represents the relative capacity of seedlings to maintain biomass accumulation under drought stress during the early growth stage. PC3, with an eigenvalue of 2.02 and a contribution rate of 13.47%, exhibited a high positive loading on GEI, corresponding to the germination capacity of maize seeds; in contrast, PLI and FWPI had high negative loadings on this component, This suggests that PC3 captures the physiological relationship, or potential trade-off, between initial germination vigor (GEI) and early shoot development (PLI, FWPI) under water-deficit conditions.PC4 and PC5, with eigenvalues of 1.38 and 1.04 and contribution rates of 9.22% and 6.94% respectively, also played important roles. PC4 had high loadings on RLI and PHI, representing seedling growth capacity, particularly early root architecture and shoot elongation, in response to limited water availability. This reflects the plant’s ability to maintain critical morphological structures for resource acquisition (e.g., water and light). PC5 was primarily defined by a high positive loading on ChlI, indicating its association with photosynthetic capacity in maize seedlings. Together, these five PCs explained 75.22% of the total variance, effectively capturing the majority of information from the 15 traits and representing the variation in maize germination and seedling development under drought stress.

**Table 6:** Eigenvector values, contribution rates, and cumulative contribution rates of principal components.

Trait	PC1	PC2	PC3	PC4	PC5
GRI	0.54	0.29	0.62	-0.24	0.01
GPI	0.48	0.15	0.74	-0.30	0.04
GII	0.64	-0.01	0.51	0.03	0.03
RLI	0.63	-0.32	0.09	0.46	-0.12
PLI	0.68	0.00	-0.50	-0.23	0.08
FWPI	0.80	0.06	-0.50	-0.24	0.03
FWRI	0.70	-0.32	0.02	0.41	-0.22
DWPI	0.82	0.16	-0.33	-0.21	0.02
NRI	0.64	-0.02	-0.09	0.31	0.26
PHI	0.11	0.32	-0.21	0.47	-0.34
CHLI	-0.06	-0.02	0.04	0.42	0.85
SFWI	-0.02	0.39	0.32	0.41	-0.22
RFWI	-0.07	0.88	-0.09	0.19	0.03
SDWI	0.04	0.82	0.07	-0.05	0.12
RDWI	0.03	0.87	-0.24	0.02	-0.03
Eigenvalue	4.03	2.81	2.02	1.38	1.04
Contribution rate (%)	26.89	18.70	13.47	9.22	6.94
Cumulative contribution rate (%)	26.89	45.59	59.06	68.28	75.22

I, drought resistance index; PL, plumule length; RL, radicle length; NR, number of radicles; FWP, plumule fresh weight; FWR, radicle fresh weight; DWP, plumule dry weight; SFW, shoot fresh weight; RFW, root fresh weight; SDW, shoot dry weight; RDW, root dry weight; GR, germination rate; GP, germination energy; GI, germination index; PH, plant height; Chl, chlorophyll content.

### 3.7 Comprehensive evaluation of drought resistance in maize hybrids

Membership function values were calculated based on the drought resistance indices of 15 traits, and weights were assigned by integrating the results of PCA. A comprehensive score, the D-value (Equations 6–8), was then obtained and used to rank all varieties from highest to lowest (**Table 7**). The D-values ranged from 0.206 to 0.647. Variety V30 ranked first, with a D-value of 0.647, followed closely by V34 (0.632), indicating that these two varieties exhibited superior performance within the drought tolerance evaluation system. In contrast, V5 had the lowest D-value (0.206), ranking 41st, suggesting relatively weak drought resistance.

**Table 7:** Comprehensive evaluation of drought resistance.

Variety	D values	Ranking	Variety	D values	Ranking
V30	0.647	1	V11	0.440	22
V34	0.632	2	V36	0.433	23
V8	0.626	3	V32	0.421	24
V28	0.619	4	V12	0.421	25
V31	0.601	5	V25	0.420	26
V14	0.593	6	V2	0.412	27
V10	0.583	7	V26	0.399	28
V20	0.555	8	V37	0.397	29
V24	0.554	9	V16	0.387	30
V22	0.544	10	V3	0.368	31
V35	0.544	11	V40	0.355	32
V29	0.524	12	V38	0.351	33
V1	0.507	13	V39	0.343	34
V23	0.498	14	V19	0.323	35
V18	0.478	15	V4	0.319	36
V33	0.472	16	V41	0.311	37
V21	0.462	17	V17	0.303	38
V15	0.461	18	V6	0.259	39
V27	0.458	19	V9	0.226	40
V13	0.458	20	V5	0.206	41
V7	0.455	21			

### 3.8 Cluster analysis of D-values among different maize varieties

**Figure 5:**
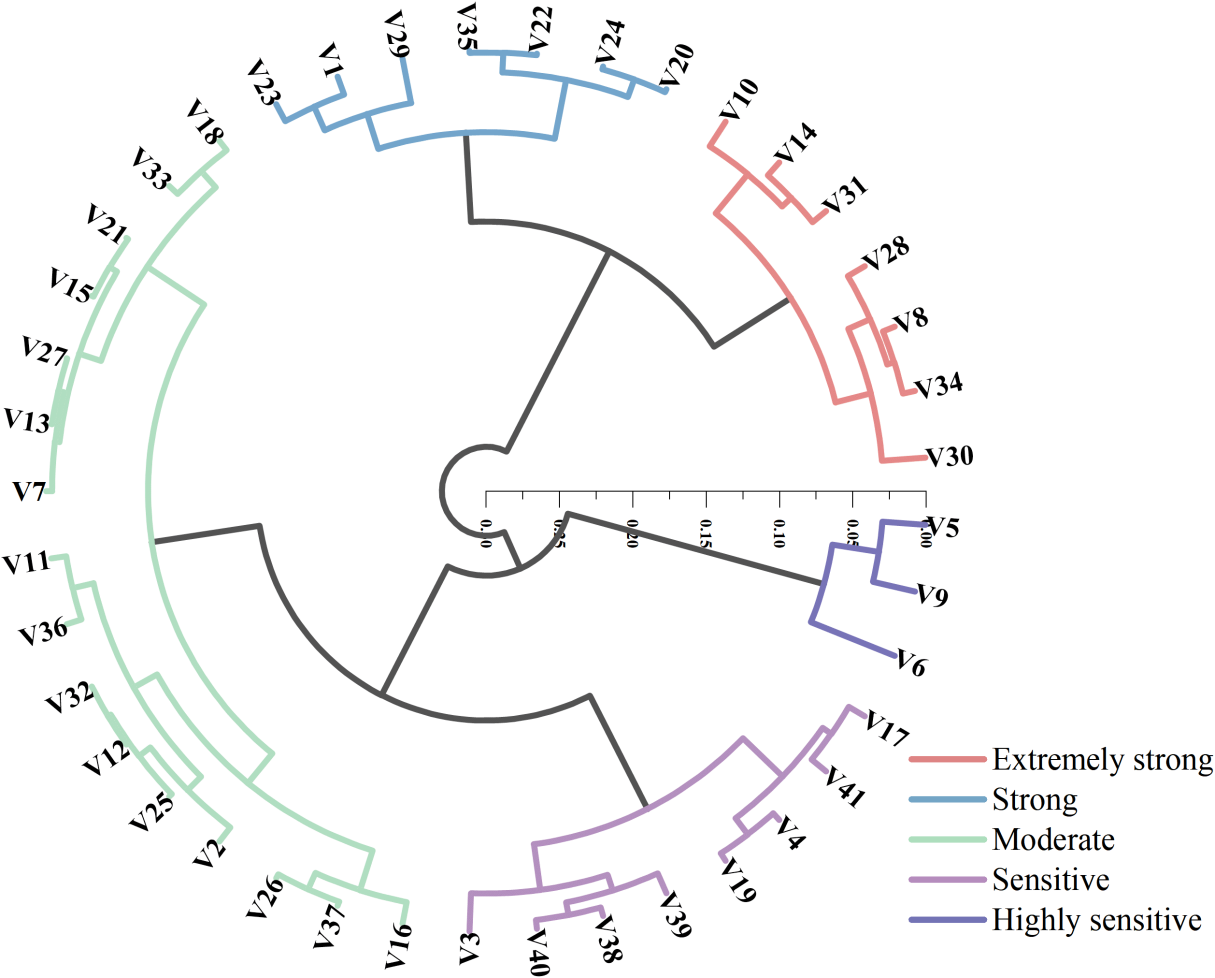
Cluster analysis of D-values for 41 maize varieties.

Cluster analysis based on D-values was conducted to classify the 41 maize varieties into five distinct drought resistance levels (**Figure 5**), using a Euclidean distance threshold of 0.1. The five categories were defined as: extremely strong drought resistance, strong drought resistance, moderate drought resistance, drought sensitivity, and high drought sensitivity. Among the varieties, seven were categorized as having extremely strong drought resistance, with D-values ranging from 0.58 to 0.65, accounting for 17% of the total. Another seven varieties (17%) fell into the strong drought resistance group, with D-values between 0.50 and 0.55. Moderate drought resistance was observed in 16 varieties, with D-values ranging from 0.39 to 0.48, representing the largest group at 39%. Eight varieties (20%) were classified as drought-sensitive, with D-values between 0.30 and 0.37. The remaining three varieties (7%) were assigned to the high drought sensitivity group, with D-values ranging from 0.21 to 0.26. These three highly sensitive varieties exhibited the lowest drought tolerance in the dataset, suggesting that their growth and development are likely to be severely inhibited under drought conditions.

### 3.9 Visualization of two-dimensional distribution and trait associations based on PCA

**Figure 6:**
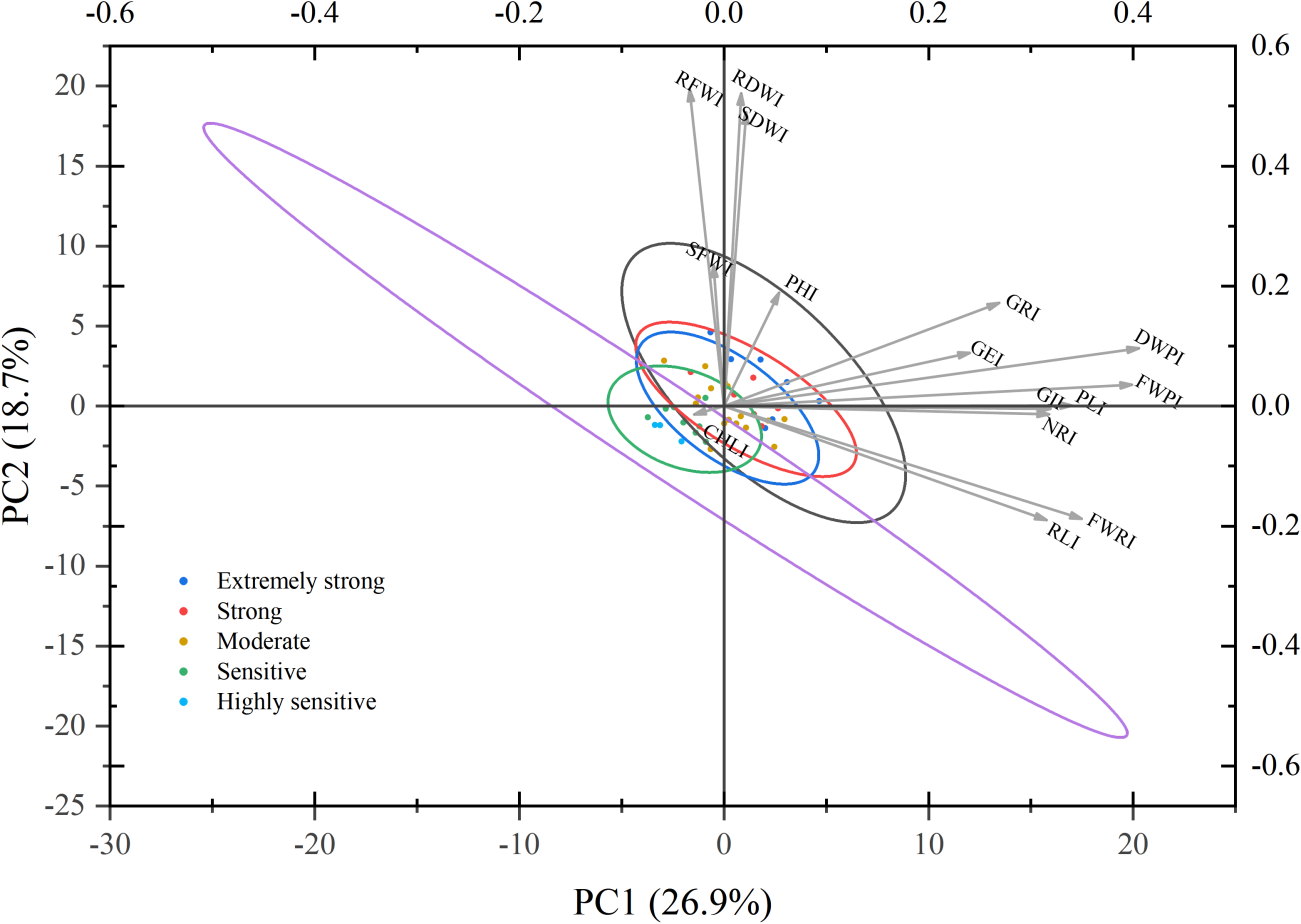
Principal component analysis plot of different maize varieties and drought resistance indices. This figure displays a biplot (PC1 vs. PC2) from the PCA of drought resistance indices for 15 traits across 41 maize germplasms. Each data point represents a distinct maize variety, with colors indicating drought resistance levels as classified by D-value cluster analysis: dark blue represents extremely strong drought resistance; blue, strong drought resistance; green, moderate drought resistance; orange, drought sensitivity; and light blue, high drought sensitivity. The ellipses illustrate the distribution ranges (e.g., covariance) of the data points within each drought resistance category. Vector arrows indicate the direction and relative magnitude (based on arrow length) of various drought resistance trait indices (I) in the principal component space. The trait abbreviations are defined as follows: I, drought resistance index; PL, plumule length; RL, radicle length; NR, number of radicles; FWP, plumule fresh weight; FWR, radicle fresh weight; DWP, plumule dry weight; SFW, shoot fresh weight; RFW, root fresh weight; SDW, shoot dry weight; RDW, root dry weight; GR, germination rate; GP, germination energy; GI, germination index; PH, plant height; Chl, chlorophyll content.

To explore the distribution patterns of maize germplasms with varying drought resistance levels and their associations with key drought resistance indices, PCA was performed using the drought resistance indices of 15 traits. The results were visualized in a two-dimensional phenotypic space (**Figure 6**). The first two PCs, PC1 and PC2, accounted for 26.89% and 18.70% of the variance, respectively, with a cumulative contribution rate of 45.59%. This indicates that the major proportion of variation in comprehensive drought resistance during the germination and seedling stages under drought stress was effectively captured. Among the traits, the dry weight preservation index (DWPI) and fresh weight preservation index (FWPI) emerged as the primary phenotypic features distinguishing strongly drought-resistant germplasms from those with weak drought resistance. Additionally, the GRI and GE index (GEI) were identified as key contributors to PC2, significantly influencing the classification of drought resistance levels.

### 3.10 Screening of drought resistance indices and establishment of regression models at maize germination and seedling stages

Stepwise regression analysis was employed to identify key drought resistance traits in maize, establish a predictive model for drought tolerance, and determine the independent variables with the greatest influence on the dependent variable. This approach enables effective prediction of drought resistance across different maize varieties. As the D-value integrates multiple drought resistance indices, it serves as a comprehensive measure of a variety’s overall performance under drought stress. Therefore, stepwise regression was conducted using the D-value as the dependent variable and the drought resistance indices of eight traits, GPI, RLI, FWPI, DWPI, plant height index (PHI), Chl index (CHLI), RFWI, and RDWI, as independent variables, all of which exhibited high loadings in the PCA. The optimal regression equation obtained was: D = -0.002 + 0.13 × GPI + 0.938 × RFWI + 0.115 × RLI + 0.112 × DWPI + 0.097 × CHLI. The model demonstrated a high goodness of fit, with a coefficient of determination (R²) of 0.856 and an adjusted R² of 0.835. The equation reached an extremely significant level (p < 0.01), indicating strong predictive power for evaluating drought resistance during the germination and seedling stages. These results suggest that GPI, RFWI, RLI, DWPI, and CHLI can serve as core indicators for screening and identifying drought-resistant maize varieties.

## 4 Discussion

### 4.1 Trait responses of maize to drought stress at germination and seedling stages

Drought-induced abiotic stress inhibits radicle and plumule growth during maize germination, thereby impairing seedling vigor, development, and overall growth, ultimately leading to reduced productivity and yield (Jian et al., 2016; Kim and Lee, 2023). In this study, drought conditions simulated by 20% PEG - 6000 significantly reduced GR, GE, RL, and PL during the germination stage. Additionally, seedling height, Chl, and biomass accumulation also showed declining trends. These findings are consistent with the results of (Efeoğlu et al., 2009) in maize seedlings and (Tripathi et al., 2024) during germination, and align with the PEG concentration-dependent inhibition reported by (Mustamu et al., 2023) in Indonesian local maize varieties. Similarly (Queiroz et al., 2019), observed reductions in both shoot and root dry matter with increasing PEG concentrations under drought stress.

Chlorophyll plays a central role in converting light energy into chemical energy during photosynthesis; its content is a direct indicator of photosynthetic capacity (Lu et al., 2024). Drought stress impairs photosynthetic function through two main mechanisms: a reduction in CO_2_ assimilation efficiency (Efeoğlu et al., 2009) and inhibition of chlorophyll biosynthesis (Zahra et al., 2023). Declining Chl reduces ATP and NADPH production during the light-dependent reactions of photosynthesis, thereby limiting carbohydrate synthesis and transport. This ultimately restricts seed germination and early seedling growth in maize (Reddy et al., 2004).

Notably, the coefficients of variation for most traits increased significantly under drought stress, indicating substantial genotypic variability in drought responses among maize cultivars. This observation supports findings by (Badr et al., 2020), who reported differential drought responses across maize genotypes under simulated stress, underscoring the extensive genetic diversity for drought tolerance within maize germplasm. Root-related traits showed particularly pronounced variation. While drought stress markedly suppressed radicle and plumule elongation in the present study, Chen et al (CaiJin et al., 2025). documented radicle elongation in alfalfa under similar conditions, suggesting fundamental differences in drought adaptation strategies between monocotyledonous and dicotyledonous species.

### 4.2 Screening of drought resistance indices for maize germplasm resources at germination and seedling stages

Screening drought resistance indicators during the germination and seedling stages is essential for identifying drought-tolerant maize germplasm (Joshi et al., 2011). As drought resistance is a complex polygenic quantitative trait, accurate evaluation requires the scientific selection of core indicators (WU and BAO, 2012; Li et al., 2023). Multivariate statistical methods enable the quantification of drought-related traits and the establishment of their quantitative relationships with overall drought resistance, thereby improving screening efficiency. Correlation analysis helps elucidate inter-trait associations, guiding the selection of key indicators (Crevelari et al., 2018).

In this study, significant correlations were observed between PDW, RDW, and the comprehensive DRI (D-value), underscoring the critical role of seed reserve mobilization in mitigating drought stress during germination. These findings reinforce that germination capacity and root performance are pivotal traits for drought resistance throughout early maize development, consistent with the results of (Xianbin et al., 2024), who identified DWP and RFW as sensitive indicators for drought tolerance. The RLI showed significant positive correlations with the GI (GII), nodal root index (NRI), and PFWI, indicating that genotypes with longer radicles under drought stress also demonstrated higher germination vigor, increased root number, and greater plumule biomass (Badr et al., 2020). Moreover, the strong positive correlations between the RFWI and both RDWI and SDWI highlight root biomass accumulation as a key determinant of overall drought resistance. Genotypes that sustain higher biomass under stress typically exhibit greater drought tolerance (Poorter et al., 2011).

PCA consolidated 15 drought-related indices into five uncorrelated PCs, with a cumulative contribution of 75.22%, effectively summarizing trait variation (Wang and Peng, 2021; Zhang et al., 2024). DWPI, RFWI, GEI, PHI, and CHLI were identified as core indicators. PC1 and PC3 were predominantly loaded by DWPI and GEI, respectively, highlighting the importance of plumule dry matter reserves and GE in conferring drought tolerance during germination. Similarly, Xianbin et al (Crevelari et al., 2018). identified DWP and GE as sensitive traits for drought tolerance in maize. PC2 and PC4 were mainly associated with RFWI and PHI, reflecting a common drought adaptation strategy involving increased resource allocation to root biomass for improved water uptake. The importance of RFW and PH for drought resistance was also validated by (Shahzad et al., 2022) in maize genotypes. PC5 emphasized Chl as a key indicator of photosynthetic performance under drought stress, consistent with (LI et al., 2006) in barley and other crops (Liu et al., 2015). also reported declines in GR, GE, shoot fresh weight (SFW), and RFW under drought stress, further supporting the selection of DWPI, RFWI, GEI, PHI, and CHLI as core drought resistance indicators. Methodologically, this study aligns with the PCA-based drought evaluation model proposed by (Sun et al., 2021) for cotton.

Stepwise regression analysis yielded a predictive equation (D = −0.002 + 0.13×GEI + 0.938×RFWI + 0.115×RLI + 0.112×DWPI + 0.097×CHLI), with a high coefficient of determination (R² = 0.856), indicating strong predictive capability. The results demonstrate that five indices, GEI, RFWI, RLI, DWPI, and CHLI, are reliable predictors of comprehensive drought resistance. This screening strategy is similar to the multivariate regression model proposed by (Saed-Moucheshi et al., 2013) for yield-related traits in wheat, although crop-specific adaptations and trait selection criteria may lead to differences in indicator performance.

### 4.3 Significance of identification and selection of drought-resistant maize germplasm resources

In this study, the comprehensive evaluation value (D-value), calculated using the membership function method, integrated multi-trait drought resistance information and effectively reflected the overall performance of maize under drought stress (Sun et al., 2021; Wang et al., 2023). This approach is consistent with methodologies employed by (Liu et al., 2015) in wheat addition lines and by Cheng et al (Wang and Peng, 2021). in the germination-stage drought resistance evaluation of rapeseed, thereby validating the reliability of the D-value for comprehensive drought tolerance assessment across crops. By combining D-values with cluster analysis, 41 maize germplasms were successfully classified into five drought resistance categories, and seven highly drought-resistant varieties were identified, Yuanyuan 1, Ximon 6, Jinongyu 309, Nongkeyu 368, Xuanhe 8, Hengyu 369, and Nongfu 99 (Shahzad et al., 2022). demonstrated that phenotypic screening at the seedling stage can effectively predict yield stability at maturity, indirectly supporting the potential applicability of these selected germplasms in arid and semi-arid regions.

The comprehensive evaluation system developed in this study, integrating multidimensional traits from both the germination and seedling stages, along with multivariate statistical analyses (correlation analysis, PCA, regression analysis, and cluster analysis) and the membership function method, overcomes the limitations of traditional screening methods based on single or few indicators (WU and BAO, 2012; Liu et al., 2023). This system offers a more holistic, objective, and efficient protocol for early-stage drought resistance evaluation of maize germplasm. Furthermore, breeders may selectively utilize parental lines exhibiting robust embryonic plumules (high DWP), strong radicle elongation (high RL), or well-developed root systems (high RFW). Alternatively, early-generation selection could prioritize individuals with sustained chlorophyll retention (high Chl) and rapid germination (high GE) under stress conditions.

The methodological framework established here is also applicable to drought resistance research in other crops, such as sorghum and millet, offering a standardized workflow for germplasm evaluation in drought-prone environments. Future studies should incorporate single-cell transcriptomic analyses to advance drought resistance breeding from phenotypic selection to molecular design. Broadening investigations across the germination and seedling stages of diverse maize germplasms will aid in identifying genotypes with superior drought-resistance alleles, thereby providing foundational resources for molecular breeding aimed at enhancing maize resilience under water-deficit conditions.

## 5 Conclusion

Drought stress markedly inhibited maize seed germination, radicle and plumule elongation, seedling development, chlorophyll synthesis, and biomass accumulation. Based on the comprehensive DRI (D-value), the 41 tested germplasms were categorized into five drought resistance levels: extremely strong, strong, moderate, sensitive, and highly sensitive. Seven varieties, Yuanyuan 1, Ximon 6, Jinongyu 309, Nongkeyu 368, Xuanhe 8, Hengyu 369, and Nongfu 99, were identified as extremely drought-resistant. Key traits identified for early-stage drought resistance evaluation included PDW, RFW, GE, plant height, and Chl. A predictive regression model was established to assess drought resistance: D = -0.002 + 0.13×GPI + 0.938×RFWI + 0.115×RLI + 0.112×DWPI + 0.097×CHLI, demonstrating high accuracy in estimating drought resistance at both germination and seedling stages. This study provides a scientific basis for the identification of drought-tolerant maize genotypes, supports the screening of elite drought-resistance alleles, and offers foundational germplasm resources for future drought-resilient maize breeding programs.

## Data Availability

The original contributions presented in the study are included in the article/supplementary material. Further inquiries can be directed to the corresponding author.
